# Revelation of Different Nanoparticle-Uptake Behavior in Two Standard Cell Lines NIH/3T3 and A549 by Flow Cytometry and Time-Lapse Imaging

**DOI:** 10.3390/toxics5030015

**Published:** 2017-07-19

**Authors:** André Jochums, Elsa Friehs, Franziska Sambale, Antonina Lavrentieva, Detlef Bahnemann, Thomas Scheper

**Affiliations:** 1 Institute of Technical Chemistry, Gottfried Wilhelm Leibniz Universität Hannover, 30167 Hannover, Germany; jochums@iftc.uni-hannover.de (A.J.); friehs@iftc.uni-hannover.de (E.F.); sambale@iftc.uni-hannover.de (F.S.); Bahnemann@iftc.uni-hannover.de (D.B.); scheper@iftc.uni-hannover.de (T.S.); 2 Laboratory “Photoactive Nanocomposite Materials” (Director), Saint-Petersburg State University, 198504 Saint-Petersburg, Russia

**Keywords:** nanoparticle-uptake, titanium dioxide, flow cytometry, light scatter, fluorescence labeling, time lapse imaging

## Abstract

The uptake of nanomaterials into different cell types is a central pharmacological issue for the determination of nanotoxicity as well as for the development of drug delivery strategies. Most responses of the cells depend on their intracellular interactions with nanoparticles (NPs). Uptake behavior can be precisely investigated in vitro, with sensitive high throughput methods such as flow cytometry. In this study, we investigated two different standard cell lines, human lung carcinoma (A549) and mouse fibroblast (NIH/3T3) cells, regarding their uptake behavior of titanium dioxide NPs. Cells were incubated with different concentrations of TiO_2_ NPs and samples were taken at certain time points to compare the uptake kinetics of both cell lines. Samples were analyzed with the help of flow cytometry by studying changes in the side and forward scattering signal. To additionally enable a detection via fluorescence, NPs were labeled with the fluorescent dye fluorescein isothiocyanate (FITC) and propidium iodide (PI). We found that NIH/3T3 cells take up the studied NPs more efficiently than A549 cells. These findings were supported by time-lapse microscopic imaging of the cells incubated with TiO_2_ NPs. Our results confirm that the uptake behavior of individual cell types has to be considered before interpreting any results of nanomaterial studies.

## 1. Introduction

Nanotechnology is an intensively developing field where the nanomaterials used in various products have an increasing impact on the consumers. This leads to more careful evaluation of the nanomaterials’ safety. Among others, there is a big interest in if and how fast nanoparticles (NPs) are taken up by mammalian cells. The importance of this information relies on the possible occasional toxic effects, but also on medical applications of NPs for e.g., drug delivery [1] or detection systems. The rapid development of new nanomaterials requires fast and cheap cell-based tests, which enable the analysis of possible toxic effects as well as their potential for pharmaceutical applications. Many newly-developed drugs have been withdrawn during animal trials, because the cell-based assays were not able to detect the hazards [2]. Flow cytometry (FCM) is a well-established and highly efficient technology for high throughput cell analysis [3,4,5]. Sample preparation for FCM is fast and simple, especially in comparison to other established techniques such as transmission electron microscopy (TEM) [6]. A flow cytometer can analyze around thousand cells per second, whereby each cell is separated and analyzed individually [7]. For each cell, the fluorescence and the light scattering signals are detected and can provide information about the cellular effects that occur during the exposure to NPs. The side scattered (SSC) light is measured at a 90° angle and correlates with the internal granularity of the cells. It has been shown that the SSC correlates with the concentration of attached or internalized NPs [8,9,10,11,12]. Investigation of the light scattering signal is advantageous, because fluorescent tags need additional preparation steps, which can influence the particle properties and behavior [13]. However, changes of the scattering signal can be induced by different factors, such as changes in the cell morphology and/or viability. Hence, fluorescence based assays could be more robust in order to avoid wrong interpretations. FCM makes it possible to detect the internalized nanoparticles and to simultaneously indicate potential toxic effects with easy and fast sample preparation, high throughput and statistically relevant results [14].

In this study, we show the potential of flow cytometric analysis for fast investigation of uptake behavior of titanium dioxide (TiO_2_) NPs in two different cell lines: A549 and NIH/3T3. TiO_2_ NPs are chemically inert particles that are used in numerous everyday consumer products, including food. Therefore, the hazards and cellular effects of these NPs have been recently re-evaluated and discussed [15,16,17,18,19]. In addition to FCM studies, time-lapse microscopy of cells treated with NPs was performed. To support the results determined by the side scattering signal, a simple labeling method to additionally detect the NPs by fluorescence was used.

## 2. Materials and Methods 

### 2.1. Cell Culture

NIH/3T3 mouse fibroblast cells (DMSZ No.: ACC 59) and A549 human lung carcinoma cells (DSMZ No.: ACC 107) were purchased from the German Collection of Microorganisms and cell cultures (DSMZ). Both cell lines were cultivated in Dulbecco’s Modified Eagle’s Medium (DMEM) (D7777 Sigma-Aldrich, Steinheim, Germany) supplemented with 10% fetal calf serum (FCS) and 100 µg mL^−1^ antibiotics (penicillin-streptomycin) in a humidified environment at 37 °C and 5% CO_2_. Approximately every three days the cultures reached 70–80% confluence and the cells were sub-cultivated. All used cells had a passage number less than 20.

### 2.2. Nanoparticles

In this study, titanium dioxide NPs (TiO_2_) with a primary particle size <10 nm and pure anatase phase have been used (Hombikat UV100), kindly provided by Sachtleben GmbH, Duisburg, Germany [20]. The small, semispherical NPs usually congregate to larger, fast-sedimenting aggregates of up to 3–5 µm diameter (Figures S1 and S2). 

### 2.3. FITC-Labeling of Titanium Dioxide Nanoparticles

For fluorescent labeling of TiO_2_ NPs, 200 µL of a 10% UV100 suspension in ethanol/methanol were resuspended in 500 µL fluorescein isothiocyanate (FITC) solution (5 mg mL^−1^ in ethanol, Sigma Aldrich) and the suspension was shaken in the dark at 1000 rpm at room temperature overnight (Eppendorf^®^ Thermomixer^®^). Particles were separated via centrifugation at 7000 rcf and washed several times with sterile ddH_2_O until the supernatant turned colorless. The obtained FITC-TiO_2_ NPs were resuspended in phosphate-buffered saline (PBS) to a final concentration of 1 mg mL^−1^ and stored in the dark at 4 °C until use. 

### 2.4. Exposure to Cells with Titanium Dioxide Nanoparticles

To provide a sufficient number of cells for FCM analysis, 200,000 NIH/3T3 cells per well or 300,000 A549 cells per well were cultivated in 2 mL medium in 6-well plates (Sarstedt AG & Co, Nümbrecht, Germany) for 24 h in a humidified environment at 37 °C and 5% CO_2_ until a 70–80% confluence was reached. Afterwards, the medium was completely removed and replaced with fresh medium containing different concentrations of FITC-TiO_2_ NPs (0, 5, 10, 15, 20, 25 µg mL^−1^). Low concentrations were chosen because of the strong scattering of TiO_2_ NPs. Moreover, by using a low concentration of NPs the sensitivity of the method could be evaluated. The exposure was carried out for 24 h in an incubator at 37 °C and 5% CO_2_. In the second approach, the exposure time was varied (0, 0.5, 1, 2, 4, 6, 8, or 24 h) with a constant NP concentration of 12.5 µg mL^−1^. After the exposure time, the cells were prepared for FCM analysis. For experiments with propidium iodide (PI), unlabeled UV100 TiO_2_ NPs were used. 

### 2.5. Flow Cytometry and Sample Preparation

After cultivation and exposure to NPs, cells were washed once with PBS, detached with 500 µL Accutase^®^ (Sigma-Aldrich, Steinheim, Germany) and centrifuged at 300 rcf for 5 min. Then the cells were washed twice with PBS. Finally, they were resuspended in 500 µL PBS and forward scattering (FSC), side scattering (SSC) and fluorescence signals were measured via flow cytometry. For experiments with PI, cells were resuspended in 100 µL of a buffer solution (10 mM HEPES (pH 7.4), 0.14 M NaCl and 2.5 mM CaCl_2_) containing 5 µL PI (1 mg mL^−1^, Sigma-Aldrich) after washing. The incubation was carried out at room temperature for 15 min and 400 µL of buffer were added before flow cytometric analysis was performed. 

The samples were analyzed by a BD Accuri C6 (Becton Dickinson, Franklin Lakes, NJ, USA) with a 488 nm Argon-ion laser. Red fluorescence light of PI was collected by a 670 long-pass filter. FITC fluorescence was collected by a 533/30 band-pass filter. At least 10,000 events per sample were analyzed. The data were analyzed with BD Accuri C6 Software (v. 1.0, Becton Dickinson, Franklin Lakes, NJ, USA, 2011). Only viable cells were taken for the analysis. The viable cell population was discriminated by forward scattered light. 

### 2.6. Time-Lapse and Fluorescence Microscopic Analysis

For time-lapse and microscopic analysis, cells were cultivated in an 8-well PCA slide (Sarstedt AG & Co, Nümbrecht, Germany) with 100,000 cells per well in 400 µL and adhered for 24 h. The medium was completely removed and 400 µL of a NP solution (12.5 µg mL^−1^) in fresh medium was added. Pictures were taken with 20× magnification every 60 s over a total imaging time of 24 h (LumaScope 600, Etaluma Inc., Carlsbad, CA, USA). For fluorescence imaging, 100,000 cells per well were cultivated on an 8-well PCA slide overnight and incubated with 400 µL of a 50 µg ml^−1^ solution of FITC-TiO_2_ NPs for 4 h. Cell nuclei were stained with 4′,6-diamidino-2-phenylindol (DAPI). Therefore, cells were washed with PBS and incubated for 15 min at 37 °C with DAPI-solution (1 µg mL^−1^ in staining buffer), then washed three times with PBS before imaging. 

## 3. Results

### 3.1. Nanoparticle Labeling with FITC

**Figure 1:**
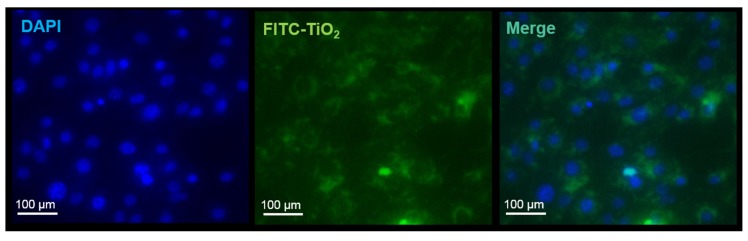
Fluorescence imaging of NIH/3T3 cells incubated with 50 µg mL^−1^ FITC-TiO_2_ NPs (green) for 4 h and stained with DAPI (blue). Magnification objective 20×.

FCM enables analysis of single cells regarding their light scattering and fluorescence signals. As unmodified TiO_2_ NPs are not fluorescent, an additional labeling step was required. Therefore, a standard fluorescence labeling molecule, FITC, was adsorbed onto the surface of the NPs. A similar labeling method has been used earlier by Feng et al. [21] to investigate the uptake of silica coated TiO_2_ NPs in nasopharyngeal carcinoma cells. After the incubation of the cells with the obtained FITC-TiO_2_ NPs, the particle aggregates were tracked by green fluorescence (Figure 1). 

FITC is a cheap yet effective labeling molecule, and excitation light sources and emission filters are usually part of the standard equipment in most laboratory fluorescence devices. However, as there is no covalent bonding of the dye on the NP surface, a decrease in the fluorescence intensity of the NPs has been observed over time, caused by possible dissociation of FITC molecules from NPs (data not shown). Therefore, FITC-TiO_2_ NPs were freshly prepared for each experiment and experiments were carried out in a time-limited manner.

### 3.2. Nanoparticle Uptake Measured by Side Scatter Signal

**Figure 2:**
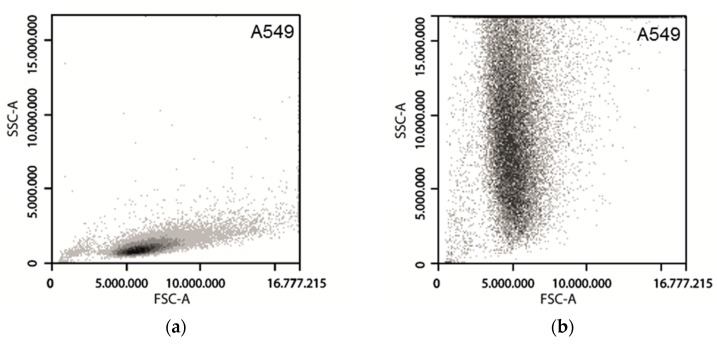

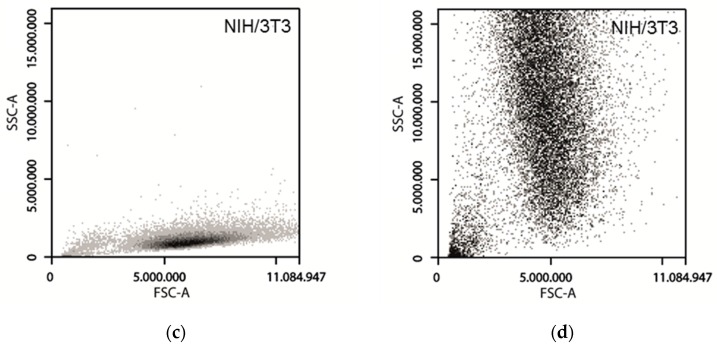
Density Plots of SSC and FSC signals of A549 and NIH/3T3 cells. Cells were incubated with each sample for 24 h. (**a**) A549 cells with 0 µg mL^−1^ FITC-TiO_2_; (**b**) A549 cells with 25 µg mL^−1^ FITC-TiO_2_; (**c**) NIH/3T3 cells with 0 µg mL^−1^ FITC-TiO_2_; (**d**) NIH/3T3 cells with 25 µg mL^−1^ FITC-TiO_2_.

The cells were analyzed by flow cytometry by acquisition of the side scattering signals after incubation with different concentrations of FITC-TiO_2_ NPs and different incubation times. A significant change in the SSC signal was revealed in treated cells in comparison to the control (Figure 2a–d). Possible toxic effects of the used NPs were evaluated by performing a cell viability assay (CellTiter Blue^®^ assay, Figure S3). Density plots of the dead controls with and without NPs can be found in the supplementary materials (Figure S4a–d). 

**Figure 3:**
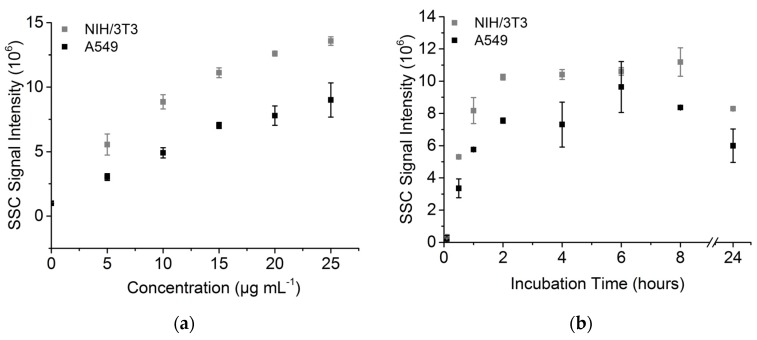
Side scatter signals of A549 and NIH/33 cells. (**a**) SSC after incubation with different FITC-TiO_2_ NP concentrations after 24 h incubation; (**b**) SSC signal kinetic of FITC-TiO_2_ NPs (12.5 µg mL^−1^).

For both cell types, treatment with different NP concentrations showed an increasing SSC signal in correlation to the FITC-TiO_2_-NP concentration (Figure 3a). The obtained concentration-to-signal correlation was linear up to 15 µg mL^−1^ for both used cell lines. NIH/3T3 cells provide a significantly higher signal (30 ± 6.5%) for each NP concentration in comparison to A549 cells. Regarding uptake kinetics, both cell lines show a high uptake rate in the first two hours of incubation. The signal intensity was constant between 2 and 8 h incubation and showed a 25% decrease between 8 and 24 h of incubation (Figure 3b). 

The decrease of the scattering signal after 24 h could be explained by a redistribution of the NPs during cell division. Since flow cytometery allows us to analyze cells individually, a direct observation of a change in the nanoparticle–cell ratio seems to be conceivable. The obtained results also indicate a higher attachment and/or uptake rate and final particle amount of TiO_2_ NPs in NIH/3T3 cells. A low uptake of NPs into A549 cells in comparison to other cell lines has been already observed by Tedja et al. [22], where H1299 cells showed a four times higher internalized amount of TiO_2_ NPs than A549 cells. 

### 3.3. Forward Scattering of Investigated Cell Lines

**Figure 4:**
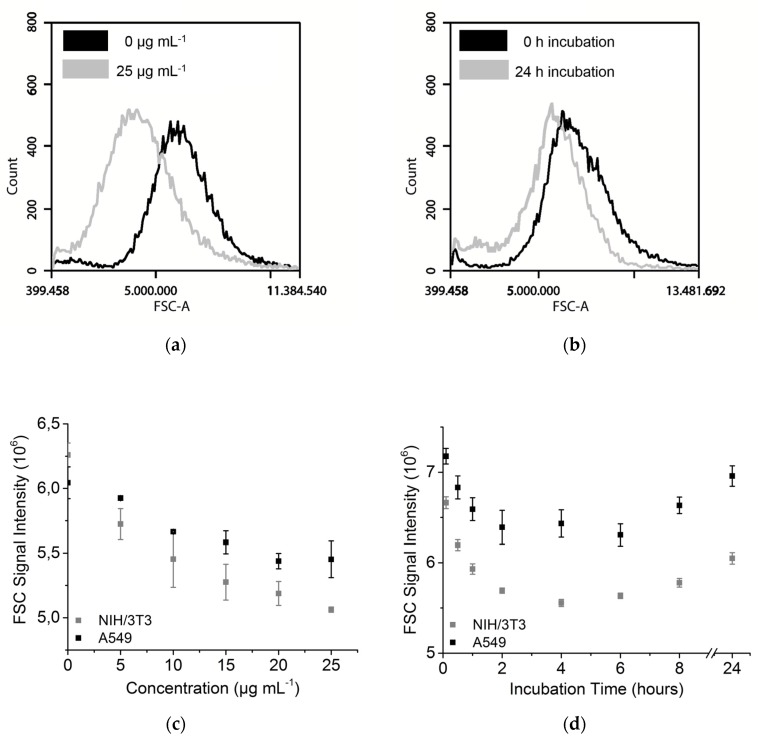
Forward scattering signal of A549 and NIH/3T3 cells. (**a**) Histogram of FSC signal with 0 µg mL^−1^ and 25 µg mL^−1^ FITC-TiO_2_ and 24 h incubation (NIH/3T3); (**b**) Histogram of FSC signal after 0 h and 24 h incubation of 12.5 µg mL^−1^ FITC-TiO_2_ (NIH/3T3); (**c**) Forward scatter after incubation with different FITC-TiO_2_ NPs concentrations (24 h incubation); (**d**) Uptake kinetic of FITC-TiO_2_ NPs (12.5 µg mL^−1^).

Forward scattered light is the light scattered with an angle of 0.5° to 5°. The forward scatter (FSC) signal shows a concentration- (Figure 4a,c) and time-dependent (Figure 4b,d) decrease in intensity. A clear shift in the FSC signal was observed (Figure 4a,b). Interestingly, the FSC signal increased after 6 h (Figure 4d). The signal intensity of A549 cells decreased by over 12% after 6 h. The NIH/3T3 cells showed a decrease of over 15% in the same time. A higher concentration of NPs led to a lower FSC signal. Signal decrease of 19% (NIH/3T3 cells) and of 10% (A549 cells) was detected at 25 µg mL^−1^ NP concentration in comparison to the untreated control. 

The FSC signal is usually used for cell size analysis and is known to be influenced by other factors, such as cell viability [23]. Live or dead discrimination with the help of PI staining showed no increase in the dead cell number (Figure S5) and the change of the forward scatter signal was too low for a typical cell death pattern. Microscopic investigations also showed no visible difference in the size before and after treatment with NPs (Section 3.5). Additionally, an increase in the signal after a longer incubation time (6 h or more) was detected, which was also observed in reverse with the side scattering signal. We assume that the decrease in the FSC signal is caused by the very strong scattering of TiO_2_ NPs, resulting in a smaller number of photons that reach the FSC detector. That would also explain the increase after longer incubation times, when the NP concentration per cell gets lower by cell division, as discussed before (Section 3.2). Figure 4a,b show that the signal shift should be considered for gating strategies. 

### 3.4. Nanoparticle Uptake Measured by Fluorescence

**Figure 5:**
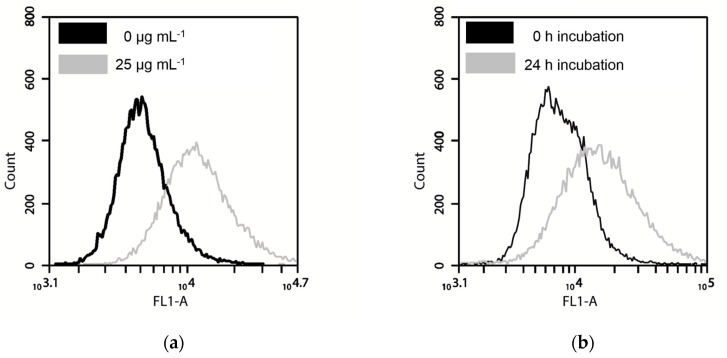

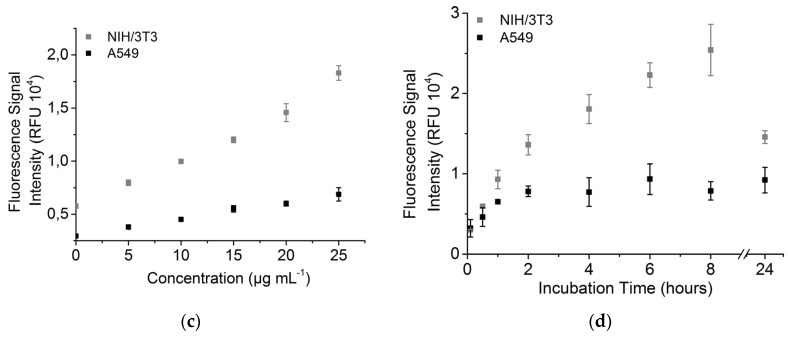
Fluorescence signal of A549 and NIH/3T3 cells. (**a**) Histogram of fluorescence signal with 0 µg mL^−1^ and 25 µg mL^−1^ TiO_2_ NPs (NIH/3T3); (**b**) Histogram of fluorescence signal after 0 h and 24 h incubation with 12.5 µg mL^−1^ FITC-TiO_2_ NPs (NIH/3T3); (**c**) Fluorescence signal after incubation with different FITC-TiO_2_ NP concentrations for 24 h; (**d**) Uptake kinetic of FITC-TiO_2_ NPs with 12.5 µg·mL^−1^, signal has been normalized to control (cells without NPs).

As the next step, NIH/3T3 and A549 cells were analyzed by FCM regarding the fluorescence properties of cells incubated with different concentrations of FITC-TiO_2_ NPs. The amount and intensity of fluorescent cells increased with increasing NP concentration and incubation times (Figure 5a,b). Both cell types showed an increased fluorescence signal in correlation to the FITC-TiO_2_-NP concentration (Figure 5c). Notably, NIH/3T3 cells showed a twice higher auto-fluorescence than A549 cells. The fluorescence signal of the NIH/3T3 cells increased rapidly with the increasing FITC-TiO_2_ concentration (3.2 times higher fluorescence signal at an NP concentration of 25 µg mL^−1^ in comparison to the control, Figure 5c), whereas the fluorescence intensity of the A549 cells increased less strongly (2.3 times higher fluorescence signal at an NP concentration of 25 µg mL^−1^ in comparison to the control, Figure 5c). Similar to the SSC signal, the fluorescence signal of the A549 cells incubated with FITC-TiO_2_ NPs increased in the first 2 h of incubation and remained constant afterwards (Figure 5d). However, it did not decrease after 24 h as observed for the SSC signal (Figure 3d). For the NIH/3T3 cells, the signal increased continuously during the time period of 8 h and showed a 36% decrease after 24 h. An increase in the fluorescence signal in the FL1 channel was not observed for unlabled UV100 particles (data not shown).

The analysis of the fluorescence signal over 24 h provides some interesting insights regarding the uptake behaviour of the cells. It has to be noticed that the data point at incubation time zero was received after incubating the cells with the NP solution for only a few seconds before preparing the samples for FCM analysis. The instant increase in the fluorescence signal compared to the control indicates a direkt attachment of NPs to the surface of the cells. The strong decrease in the signal of the NIH/3T3 cells after 24 h may arise from both the redistribution on proliferation cells and possibly the pH sensitivity of FITC. FITC looses fluorescence intensity with decreasing pH [24] and internalized NPs have been shown to end up in late endosomes or lysosomes where a pH of 4.5 and 5.5 is usual [25]. In general, the NIH/3T3 cells show a significantly higher fluorescence signal in comparison to the A549 cells, which increases strongly in correlation with particle concentration and incubation time.

### 3.5. Nanoparticle Uptake Kinetics Monitored by Microscopy

**Figure 6:**
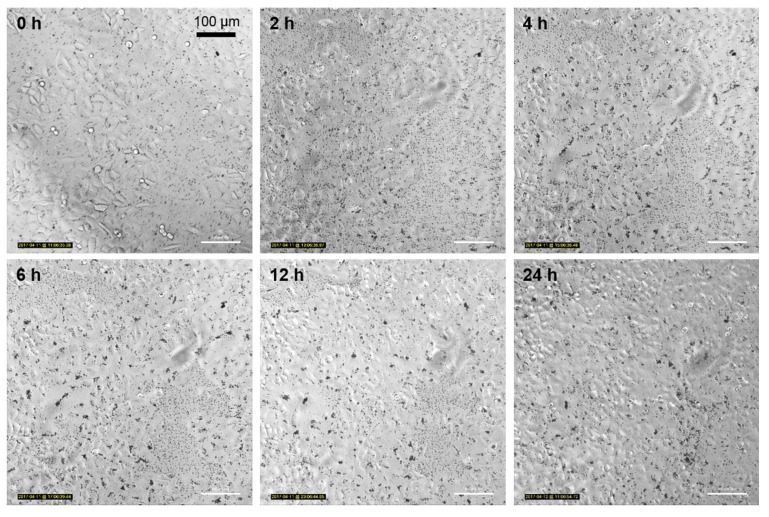
Time-lapse microscopic imaging of A549 cells incubated with 12.5 µg mL^−1^ FITC-TiO_2_ NPs for 24 h. Magnification 20×.

To support the flow cytometric results that suggest a difference in the uptake behavior of the two investigated cell lines, the cells were incubated with TiO_2_ and monitored by microscopy over 24 h. The resulting time-lapse videos of the two cell lines can be found in the supplementary materials (Video S1 and S2). We highly recommend consideration of the video material as it supports the observations described in the following chapter. In both cases, the time-lapse imaging shows a sedimentation of particle aggregates during the first hour of incubation. The aggregates clearly contrast from the cells as dark dots. In general, the NIH/3T3 cells show higher cellular mobility and proliferation rates than the A549 cells. In case of the A549 cells, the TiO_2_ NP aggregates accumulated between the cells on the cell-free surface of the culture chamber (Figure 6). Large aggregates of up to 25 nm were built after 4 h of incubation.

In the case of the A549 cells, the particles seem to be associated with the cells to a certain degree, but it remains unclear if the NP aggregates are internalized or are only attached to the cell surface. As can be seen in the time-lapse imaging, especially larger aggregates seem to detach from the cells during proliferation, therefore they were probably not taken up by the cells. Stearns et al. [26] also observed an association of ultrafine TiO_2_ NPs with the free surface of A549 rather than an actual internalization for the majority of the agglomerates. 

**Figure 7:**
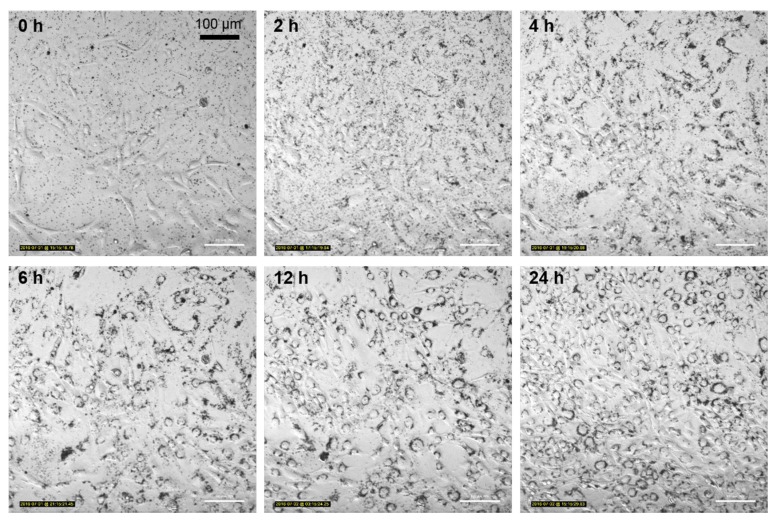
Time-lapse microscopic imaging of NIH/3T3 cells incubated with 12.5 µg mL^−1^ FITC-TiO_2_ NPs for 24 h. Magnification 20×.

In contrast, the particle aggregates showed a high affinity to the NIH/3T3 cells. After four hours, most of the particles were attached to the cells, as they move parallel to the cellular mobility and proliferation. In several cases, a random redistribution of the particles into the daughter cells during mitosis was apparent. Additionally, the particles showed a coronoid arrangement around the cell nucleus, leaving the nuclei area free. After 24 h, nearly all visible NP aggregates were localized in or on the cells and all cells showed the NP coronal in the cytoplasm (Figure 7). 

The time-lapse imaging supports the previous findings that indicate a difference in uptake behavior between the two investigated cell lines. The TiO_2_ NPs show a high affinity to the NIH/3T3 cells and seem to be internalized spontaneously with very high efficiency. The particle aggregates also show a very characteristic arrangement around the nucleus. Such an accumulation of NPs in the cytoplasm has also been observed for silver NPs [27] and iron oxide NPs [28]. It can be explained by the morphology of adherent NIH/3T3 cells. Fibroblast cells are usually flatly elongated when they are adhered to a surface, with a spherical peak that originates from the raised cell nucleus [29,30]. The cytoplasm around the nucleus provides the required physical space for large endocytosed particle aggregates. This coronal shape of NP aggregates also indicates the actual internalization of the particles, as otherwise they would be randomly distributed on the cell monolayer, without an NP-free nucleus area. 

The obtained time-lapse images also confirm the low toxicity of TiO_2_ NPs, as reported previously [31]. Even though the NIH/3T3 cells show a high content of NP aggregates in the cytoplasm, viability and proliferation activity was evidently not inhibited. The A549 cells do also not show any morphological abnormities. Although microscopic monitoring can only visualize NP aggregates and is not able to detect single NPs, it provides a closer view of the particles’ fates in the cell, since in vivo NPs also form aggregates [22]. The ratio of the suspended NPs to NP aggregates in the cells, however, must be further studied by TEM.

### 3.6. Interference with PI Signal

**Figure 8:**
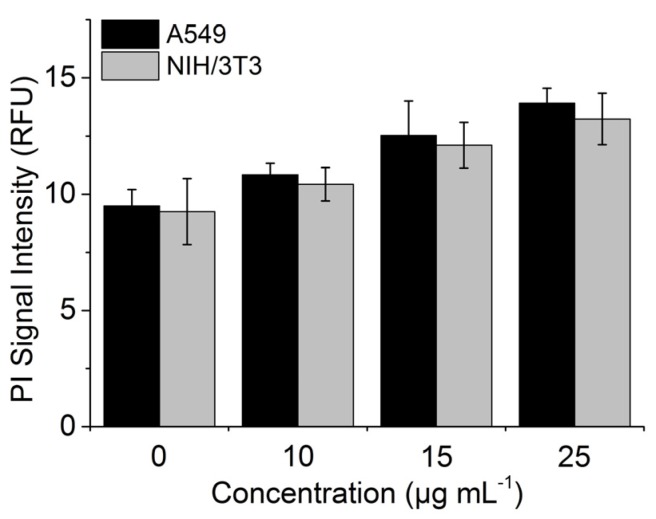
Fluorescence signal of A549 and NIH/3T3 cells after incubation with different TiO_2_ NP concentrations.

PI is a common dye for live/dead cell discrimination. It cannot pass the intact membranes of living cells, but it passes the permeable membranes of dead cells. After entering the cell, it intercalates with the DNA and the fluorescence is of up to 30-fold higher intensity [32]. We originally used PI for the evaluation of the toxic effects of our TiO_2_ NPs (there are none), but we saw an unexpected effect on the fluorescence intensity of PI. The following results show the increase of the propidium iodide signal on living cells depending on NP concentration (Figure 8). The mean PI fluorescence signal of both cell types increased by about 45% with 25 µg mL^−1^ TiO_2_ NPs, compared to no nanoparticles (dead cells excluded).

This effect has also been briefly described in the literature [32]. We made further investigations and showed the concentration dependency. Our results indicate that it might be also possible to quantify NP uptake with PI. NPs are known and used to transfer organic molecules into cells [33]. We saw the same effect with PI, the dye seems to adsorb on the surface of the TiO_2_ NPs and can be used as a reagent for uptake analysis. The signal increase needs also to be considered for gating strategies. With the used protocol, it is still easy to distinguish between living and dead cells, because the signal of dead cells is still much higher and provides a clearly separated population (Figure S5). As PI provides an increased signal when intercalated in DNA, it can act as an indicator for the uptake of TiO_2_ NPs inside the cells.

## 4. Conclusions

The invention and development of powerful and robust in vitro assays is very important for uncovering the risks and discovering the possible medical applications of nanomaterials. In this work, flow cytometry was used for the investigation of in vitro NP uptake into the cells. FCM analysis allowed us to detect differences in the uptake behavior of TiO_2_ NPs in two different cell lines. A direct correlation of the fluorescence as well as of the scattering signals of cells incubated with particles was clearly observed. The presented results additionally show how much influence the chosen cell type has on the experimental outcome. They also allowed us to receive an impression as to how different the ways of in vivo NP uptake can be, in this case via lung and connective tissue exposition. 

## Supplementary Materials

The following figures and videos are available online at www.mdpi.com/2305-6304/5/3/15/s1, Figure S1: SEM image of UV100 NPs; Figure S2: DLS measurement of UV100 NPs; Figure S3: Viability assay of NIH/3T3 and A549 cells with UV100 and FITC-TiO_2_ NPs; Figure S4: Density plots of death controls of NIH/3T3 cells; Figure S5: Histogram of PI-stained NIH/3T3 cells; Video S1: NIH/3T3 cells incubated with FITC-TiO_2_ nanoparticles for 24h; Video S2: A549 cells incubated with FITC-TiO_2_ NPs for 24h.

## References

[1] Seleci D.A., Seleci M., Jochums A., Walter J.G., Stahl F., Scheper T. (2016). Aptamer mediated niosomal drug delivery. RSC Adv..

[2] Astashkina A., Mann B., Grainger D.W. (2012). A critical evaluation of in vitro cell culture models for high-throughput drug screening and toxicity. Pharmacol. Ther..

[3] Degelau A., Freitag R., Linz F., Middendorf C., Scheper T., Bley T., Muller S., Stoll P., Reardon K.F. (1992). Immuno- and flow cytometric analytical methods for biotechnological research and process monitoring. J. Biotechnol..

[4] Moretti P., Behr L., Walter J.G., Kasper C., Stahl F., Scheper T. (2010). Characterization and improvement of cell line performance via flow cytometry and cell sorting. Eng. Life Sci..

[5] Meyer M., Scheper T., Walter J.-G. (2013). Aptamers: Versatile probes for flow cytometry. Appl. Microbiol. Biotechnol..

[6] Graham L., Orenstein J.M. (2007). Processing tissue and cells for transmission electron microscopy in diagnostic pathology and research. Nat. Protoc..

[7] Fulwyler M.J. (1965). Electronic separation of biological cells by volume. Science.

[8] Zucker R.M., Daniel K.M., Massaro E.J., Karafas S.J., Degn L.L., Boyes W.K. (2013). Detection of silver nanoparticles in cells by flow cytometry using light scatter and far-red fluorescence. Cytom. A.

[9] Suzuki H., Toyooka T., Ibuki Y. (2007). Simple and easy method to evaluate uptake potential of nanoparticles in mammalian cells using a flow cytometric light scatter analysis. Environ. Sci. Technol..

[10] Kumar A., Pandey A.K., Singh S.S., Shanker R., Dhawan A. (2011). A flow cytometric method to assess nanoparticle uptake in bacteria. Cytom. A.

[11] Toduka Y., Toyooka T., Ibuki Y. (2012). Flow cytometric evaluation of nanoparticles using side-scattered light and ROS-mediated fluorescence-correlation with genotoxicity. Environ. Sci. Technol..

[12] Zucker R.M., Massaro E.J., Sanders K.M., Degn L.L., Boyes W.K. (2010). Detection of TiO_2_ nanoparticles in cells by flow cytometry. Cytom. A.

[13] Dobrovolskaia M.A., McNeil S.E. (2007). Immunological properties of engineered nanomaterials. Nat. Nanotechnol..

[14] Lindström S. (2012). Flow Cytometry and Microscopy as Means of Studying Single Cells: A Short Introductional Overview. Methods Mol. Biol..

[15] Hamilton R.F., Wu N., Porter D., Buford M., Wolfarth M., Holian A. (2009). Particle length-dependent titanium dioxide nanomaterials toxicity and bioactivity. Part. Fibre Toxicol..

[16] Sager T.M., Kommineni C., Castranova V. (2008). Pulmonary response to intratracheal instillation of ultrafine versus fine titanium dioxide: Role of particle surface area. Part. Fibre Toxicol..

[17] Nel A., Xia T., Mädler L., Li N. (2006). Toxic potential of materials at the nanolevel. Science.

[18] Stone V., Donaldson K. (2006). Nanotoxicology: Signs of stress. Nat. Nanotechnol..

[19] Friehs E., AlSalka Y., Jonczyk R., Lavrentieva A., Jochums A., Walter J.-G., Stahl F., Scheper T., Bahnemann D. (2016). Toxicity, phototoxicity and biocidal activity of nanoparticles employed in photocatalysis. J. Photochem. Photobiol. C Photochem. Rev..

[20] Lindner M., Bahnemann D.W., Hirthe B., Griebler W.-D. (1997). Solar Water Detoxification: Novel TiO_2_ Powders as Highly Active Photocatalysts. J. Sol. Energy Eng..

[21] Feng X., Zhang S., Wu H., Lou X. (2015). A novel folic acid-conjugated TiO_2_-SiO_2_ photosensitizer for cancer targeting in photodynamic therapy. Colloids Surf. B Biointerfaces.

[22] Tedja R., Marquis C., Lim M., Amal R. (2011). Biological impacts of TiO_2_ on human lung cell lines A549 and H1299: Particle size distribution effects. J. Nanopart. Res..

[23] McGann L.E., Walterson M.L., Hogg L.M. (1988). Light scattering and cell volumes in osmotically stressed and frozen-thawed cells. Cytometry.

[24] Lanz E., Gregor M., Slavik J., Kotyk A. (1997). Use of FITC as a Fluorescent Probe for Intracellular pH Measurement. J. Fluoresc..

[25] Zhu M., Nie G., Meng H., Xia T., Nel A., Zhao Y. (2013). Physicochemical properties determine nanomaterial cellular uptake, transport, and fate. Acc. Chem. Res..

[26] Stearns R.C., Paulauskis J.D., Godleski J.J. (2001). Endocytosis of ultrafine particles by A549 cells. Am. J. Respir. Cell Mol. Biol..

[27] Caballero-Díaz E., Pfeiffer C., Kastl L., Rivera-Gil P., Simonet B., Valcárcel M., Jiménez-Lamana J., Laborda F., Parak W.J. (2013). The toxicity of silver nanoparticles depends on their uptake by cells and thus on their surface chemistry. Part. Part. Syst. Charact..

[28] Saltan N., Kutlu H.M., Hür D., Işcan A., Say R. (2011). Interaction of cancer cells with magnetic nanoparticles modified by methacrylamido-folic acid. Int. J. Nanomed..

[29] Hazel A.L., Pedley T.J. (2000). Vascular Endothelial Cells Minimize the Total Force on Their Nuclei. Biophys. J..

[30] Kim D.-H., Li B., Si F., Phillip J.M., Wirtz D., Sun S.X. (2015). Volume regulation and shape bifurcation in the cell nucleus. J. Cell Sci..

[31] Sambale F., Lavrentieva A., Stahl F., Blume C., Stiesch M., Kasper C., Bahnemann D., Scheper T. (2015). Three dimensional spheroid cell culture for nanoparticle safety testing. J. Biotechnol..

[32] Neumeyer A., Bukowski M., Veith M., Lehr C.M., Daum N. (2011). Propidium iodide labeling of nanoparticles as a novel tool for the quantification of cellular binding and uptake. Nanomed. Nanotechnol. Biol. Med..

[33] Zanin H., Hollanda L.M., Ceragioli H.J., Ferreira M.S., Machado D., Lancellotti M., Catharino R.R., Baranauskas V., Lobo A.O. (2014). Carbon nanoparticles for gene transfection in eukaryotic cell lines. Mater. Sci. Eng. C.

