# Supplementary material for: Revelation of Different Nanoparticle-Uptake Behavior in Two Standard Cell Lines NIH/3T3 and A549 by Flow Cytometry and Time-Lapse Imaging
Source: Toxics. 2017 Jul 19;5(3):15. doi: 10.3390/toxics5030015 (PMC5634699; doi:10.3390/toxics5030015)
Supplement: Supplementary file 1 [file toxics-05-00015-s001.zip › toxics-203422 supplementary.pdf]

Supplementary Material: Revelation of Different Nanoparticle-Uptake Behavior in Two Standard Cell Lines NIH/3T3 and A549 by Flow Cytometry and Time-Lapse Imaging

André Jochums, Elsa Friehs, Franziska Sambale, Antonina Lavrentieva, Detlef Bahnemann and Thomas Scheper

S1. Material and Methods

S1.1. Analysis of Hombikat UV100 Nanoparticles

Field-emission scanning electron microscopy (FE-SEM) measurement of UV100 powder was carried out on a JSM-6700F instrument (JEOL, Tokyo, Japan), using a secondary electron imaging detector (SEI) at an accelerating voltage of 2.0 kV, with working distance of 3.1 mm.

Aggregate size was additionally determined by dynamic light scattering (DLS, Malvern Instruments Ltd., zetasizer Nano ZSP, UK). Therefore, 5 μg mL^{-1} of UV100 NPs in PBS were ultra sonicated for 1 h before measurement. The instrument detected large, sedimenting, polydisperse particles around 500 nm up to 5 μm.

S1.3. Viability Assay of A549 and NIH/3T3 Cells

For analysis of toxicity of UV100 and FITC-TiO_2 NPs, 8000 cells/well (NIH/3T3) or 10,000 cells/well (A549) were seeded in 100 μL/well in 96-well plates (Sarstedt AG & Co, Nümbrecht, Germany) and cultivated overnight at 37 °C, 5% CO_2. Cells were exposed to 100 μL of a UV100 TiO_2 or FITC-TiO_2 solution (0, 5, 10, 15, 20, 25 μg mL^{-1}) for 24 h. Medium was removed and cells were incubated with 100 μL of a CellTiter Blue® reagent solution (diluted 1:10 in serum free DMEM, Promega Corp., USA) for 2 h. The resulting fluorescence signals ($544_{Ex}/590_{Em}$) were recorded with a fluorometer (Fluoroskan Ascent, Thermo Fischer Scientific Inc. USA).

S.1.4. FCM Analysis of Dead Cells

For dead controls, NIH/3T3 cells with and without NPs were heated for 5 min at 90 °C to assure killing of the cells. Cells were prepared and analyzed as described in Section 2.5.

S2. Results

S.2.1. SEM Imaging of UV100

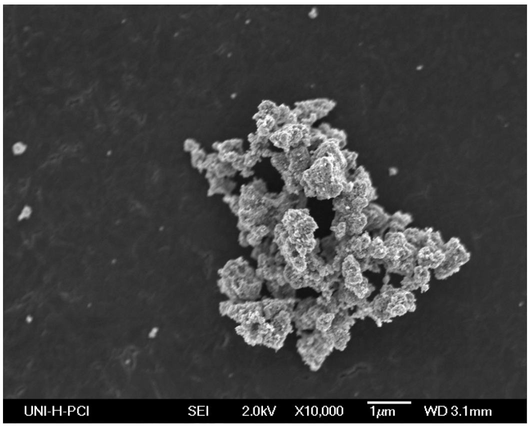

Figure S1. SEM image of UV100 NPs. Detected by secondary electron imaging (SEI) at an accelerating voltage of 2.0 kV, with working distance of 3.1 mm. 10,000× magnification.

S.2.2. DLS Measurement of UV100 NPs

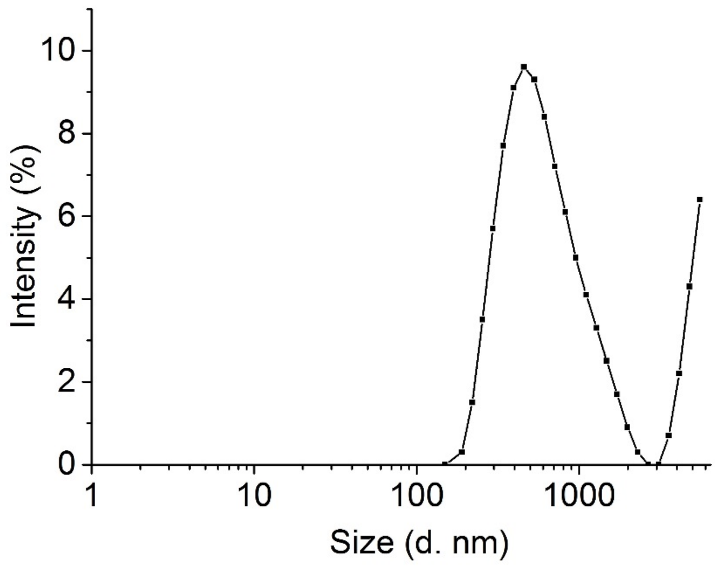

Figure S2. Intensity plot of DLS measurement of hydrodynamic size of UV100 aggregates in PBS.

S.2.3. Cell Viability Assay of NIH/3T3 and A549 Cells with TiO_2 NPs

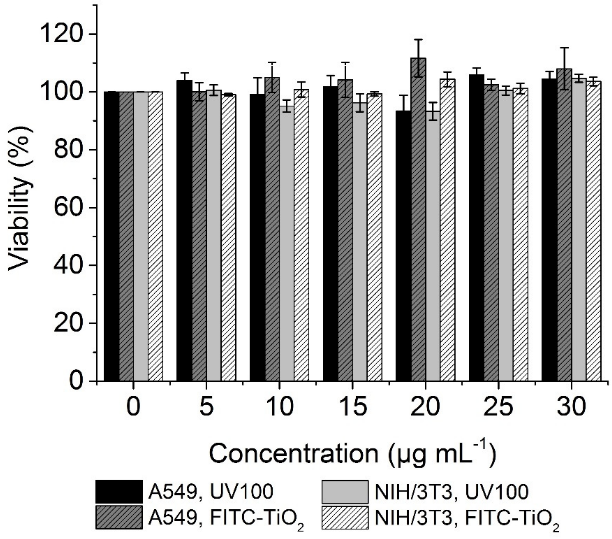

Figure S3. Cell viability of NIH/3T3 and A549 cells after TiO_2 NP incubation for 24 h. Data has been normalized to control (0 µg mL^{-1}).

S.2.4. Dead Controls of NIH/3T3 Cells Analyzed by FCM

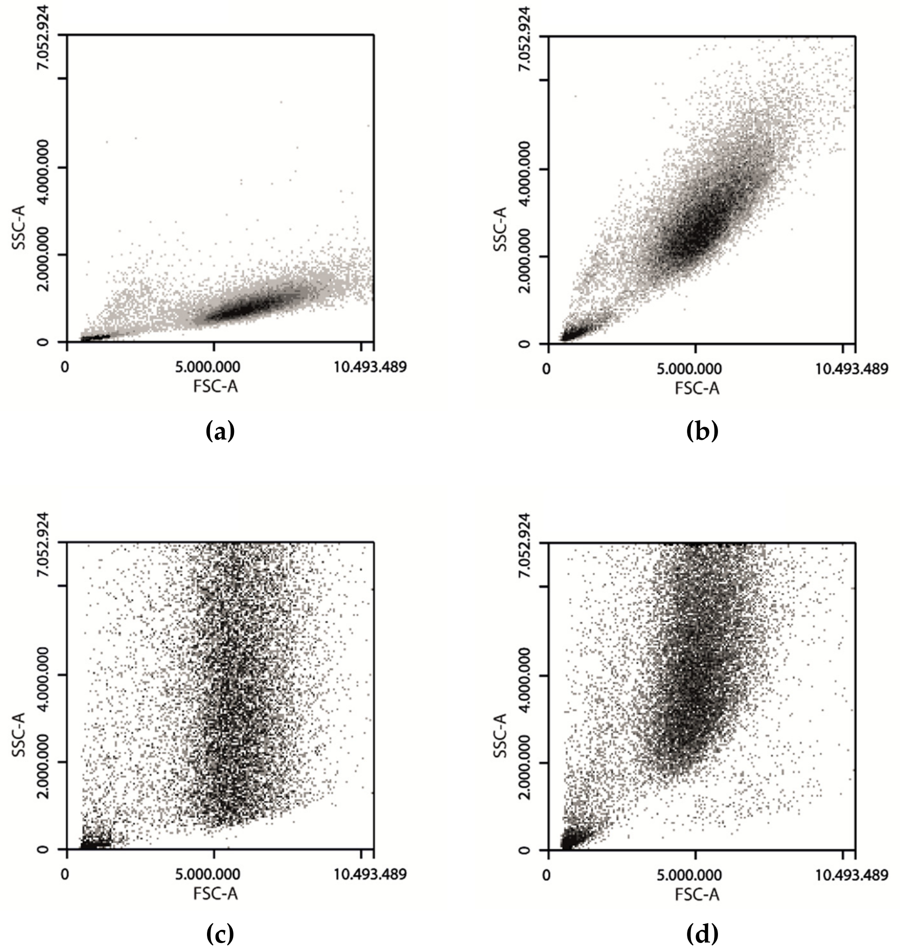

Figure S4. Density Plots of SSC and FSC signals of NIH/3T3 cells. Each sample was incubated for 24 h. (**a**) NIH/3T3 cells without UV100 NPs; (**b**) dead control of NIH/3T3 cells without NPs; (**c**) NIH/3T3 cells with 25 μg mL^{-1} UV100 NPs; (**d**) dead control of NIH/3T3 cells with 25 μg mL^{-1} UV100 NPs.

S.2.5. Dead Controls of PI-Stained NIH/3T3 Cells Analyzed by FCM

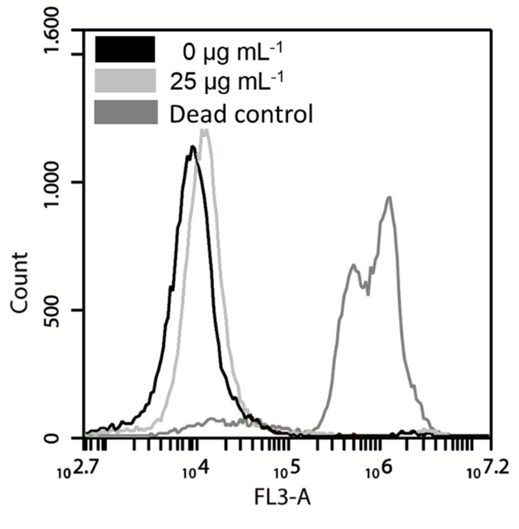

Figure S5. Histogram of PI-stained NIH/3T3 cells (live/dead) with and without UV100 NPs incubated for 24 h.
